# Multi-Type Weld Defect Detection in Galvanized Sheet MIG Welding Using an Improved YOLOv10 Model

**DOI:** 10.3390/ma19061178

**Published:** 2026-03-17

**Authors:** Bangzhi Xiao, Yadong Yang, Yinshui He, Guohong Ma

**Affiliations:** 1 School of Advanced Manufacturing, Nanchang University, Nanchang 330031, China; 15727658071@163.com; 2 Key Laboratory of Light Alloy of Jiangxi Province, Nanchang University, Nanchang 330031, China; 18334956041@163.com; 3 School of Resources Environmental and Chemical Engineering, Nanchang University, 999 Xuefu Avenue, Nanchang 330031, China; heyingshui117@163.com

**Keywords:** weld porosity detection, YOLOv10, lightweight neural network

## Abstract

Shop-floor weld inspection may appear to be a solved problem until a camera is deployed near a galvanized-sheet MIG welding line. The seam reflects light, the texture changes from frame to frame, and the defects of interest are often small and visually subtle. Additionally, the hardware near the line is rarely a data-center GPU. With those constraints in mind, this paper presents YOLO-MIG, a compact detector built on YOLOv10n for weld-seam inspection in practical production conditions. We make three focused changes to the baseline: a C2f-EMSCP backbone block to better preserve weak defect cues with modest parameter growth, a BiFPN neck to keep small-target information alive during feature fusion, and a C2fCIB head to clean up predictions that otherwise get distracted by seam edges and illumination artifacts. On a workshop-collected dataset containing 326 original images, with the training subset expanded through augmentation to 2608 labeled samples in total, YOLO-MIG achieves 98.4% mAP@0.5 and 56.29% mAP@0.5:0.95 on the test set while remaining lightweight (1.83 M parameters, 3.87 MB FP16 weights). Compared with YOLOv10n, the proposed model improves mAP@0.5 by 9.36 points and mAP@0.5:0.95 by 4.89 points, while reducing parameters, GFLOPs, and model size by 43.4%, 19.9%, and 29.9%, respectively. The results suggest that YOLO-MIG is not only accurate but also realistic to deploy at the edge for intelligent weld quality control.

## 1. Introduction

Welding defects are small imperfections with potentially serious consequences. Although severe defects such as burn-through are visually obvious, small blowholes or shallow undercuts can weaken a joint and later develop into fatigue damage. In production environments operating under tight takt times, inspection must be rapid, non-contact, and suitable for in-line use rather than for offline analysis alone. In practice, online weld quality monitoring increasingly relies on vision signals acquired during welding to meet these time constraints, and defect types such as burn-through are often treated as critical alarms in process-monitoring studies [1]. These observations highlight the need for a fast vision-based inspection pipeline that can operate continuously in production.

Classical non-destructive testing remains important, and radiographic, ultrasonic, and penetrant methods each offer specific advantages. Recent learning-based advances for ultrasonic TOFD and X-ray weld inspection have improved detection accuracy and interpretability; however, these workflows still depend on specialized setups and are not always compatible with high-throughput in-line deployment [2,3]. In practical production, safety procedures, coupling conditions, setup time, and throughput can all become bottlenecks when continuous monitoring is required. Existing studies consistently show that a method may be technically sound, yet deployment under real industrial constraints remains challenging.

That is why camera-based inspection often ends up on the shop floor. It is cheap, flexible, and easy to integrate. The catch is that galvanized-sheet MIG weld images are rarely cooperative. Vision inspection on reflective metal surfaces is notoriously sensitive to specular highlights and exposure saturation, where pseudo-defects often emerge near edges and style lines under non-uniform lighting [4]. Prior industrial metrology studies have shown that active/structured lighting and polarization-aware imaging are often required to stabilize measurements on specular metallic surfaces with large reflectivity variation [5,6,7]. The seam reflects light, texture varies, and illumination drifts across a shift. Background cues—seam boundaries, spatter-like patterns, surface scratches—can look just enough like a defect to fool a detector. Broad surveys on industrial visual inspection keep pointing to the same pain points: weak contrast, cluttered backgrounds, and distribution shifts across processes and batches.

In this work, we cast weld-seam inspection as a five-class detection problem within a seam ROI: burn-through, undercut, blowhole, embossment, and good. Deep learning makes this problem tractable. Prior work has explored weld defect analysis using segmentation, transfer learning, region proposal networks, and attention-based lightweight designs, especially for radiographic imagery. For real-time shop-floor vision, one-stage detectors in the YOLO family remain attractive: they localize and classify in a single forward pass, which is exactly what edge deployment tends to demand. Recent surveys consistently conclude that YOLO-style detectors provide one of the most favorable accuracy–latency trade-offs for weld defect detection in practical industrial settings, especially when edge deployment is required [8]. Meanwhile, weld-specific lightweight variants have explicitly targeted small-size and low-contrast defects under complex textures by enhancing multi-scale feature utilization and attention-guided heads, improving robustness without dramatically increasing model size [9,10]. Industrial datasets also frequently suffer from class imbalance and limited defect samples, motivating strategies such as transfer learning and GAN-based augmentation to improve online recognition reliability under real production conditions [11]. We start from YOLOv10n, a modern end-to-end design with a favorable accuracy–latency balance. Still, weld seams are not natural-image benchmarks. Defects are small, the background is visually messy, and naive feature fusion can wash out the weak cues you actually need. That gap is the motivation for YOLO-MIG.

Contributions:

1. We design a weld-oriented lightweight backbone component (C2f-EMSCP with a PSA-style tail) to preserve weak defect cues without bloating the model.
2. We adopt BiFPN to improve cross-scale alignment and reduce small-defect information loss during fusion.
3. We refine the prediction head using C2fCIB modules to suppress seam-edge and illumination-induced false activations.

## 2. Related Work

### 2.1. NDT and Weld Inspection Pipelines

A large part of weld inspection still rests on NDT. Radiographic and ultrasonic methods can reveal internal flaws, and penetrant testing remains useful for surface-breaking defects. In practice, radiographic testing and visual inspection are still routinely used to evaluate weld quality, often complemented by macro-/microstructural examinations to interpret defect formation and joint performance [12,13,14]. Such procedures remain effective for auditing, but they are difficult to scale into continuous, high-throughput in-line monitoring. However, deployment depends on more than physical observability: inspection speed, safety requirements, and sensitivity under changing conditions decide whether a technique fits in-line production. Modeling work and experiments show how strongly microstructure and defect morphology can influence ultrasonic responses, which complicates “set-and-forget” automation on a line. Recent learning-based analysis on NDT imagery (e.g., TOFD and X-ray) aims to accelerate interpretation and improve consistency, yet its practical deployment remains constrained by inspection setup, data stability, and throughput requirements [2,3].

### 2.2. Deep Learning for Weld Defect Analysis

Deep learning methods have been widely tested on welding defect imagery, especially radiographs. Beyond radiographs, high-speed imaging has also been used for welding process monitoring and defect-state recognition by combining visual feature extraction with machine-learning classifiers, highlighting the demand for real-time decision-making close to the production line [1]. Semantic segmentation has been used to achieve pixel-level defect delineation, and specialized deep segmentation networks report strong performance in curated settings. Surveys and recent X-ray studies report strong segmentation/detection performance on curated weld datasets, but they also emphasize dataset dependence and the difficulty of transferring models across sites and imaging conditions [3,8]. Transfer learning with pretrained CNNs can help when data is limited, which is often the case for industrial projects. In industrial environments where defect samples are scarce and class distributions are highly imbalanced, transfer learning and GAN-driven data augmentation have been explored to improve online recognition robustness [11]. Two-stage detection and region proposal-based pipelines have also been proposed for weld defect localization, offering accuracy benefits when compute budgets allow. More lightweight attention-driven networks target weld defect detection with an eye toward efficiency. Recent lightweight weld defect detectors increasingly adopt multi-scale feature enhancement and attention-guided heads to improve sensitivity to small and low-contrast defects while maintaining real-time inference on constrained hardware [9,10], reflecting a growing focus on deployable solutions rather than only offline analysis. Parallel trends appear in ultrasonic flaw detection, where attention and feature fusion improve stability across signal variations. Similarly, TOFD-oriented studies highlight the value of multi-image fusion and feature enhancement when defect cues are weak and interpretation is inherently dynamic [2].

### 2.3. Industrial Visual Defect Detection and Compact Detectors

Visual defect detection beyond welding has matured quickly, and surveys give a useful reality check: in industrial inspection of metallic surfaces, non-uniform illumination and specular highlights are repeatedly reported as major sources of pseudo-defects and unstable detection, especially around edges and textured regions [4], weak contrast and background complexity remain the dominant failure modes, while domain shift turns many “good” models into fragile ones when a new batch arrives. YOLO-style detectors are popular in industrial inspection because they are fast and straightforward to deploy, optical inspection on specular objects has explored fringe reflection and photometric-stereo-style modeling to cope with mirror-like reflectance, underscoring why appearance-based defect detection can be fragile under reflective conditions [15,16], and many works improve them via attention, feature fusion, or lightweight backbone tricks. Edge deployment adds another layer of constraints—memory footprint and GFLOPs can matter more than an extra point of mAP. Lightweight detection and efficient fusion designs for edge devices appear repeatedly in the literature, from compact backbones to multi-scale fusion strategies. EfficientDet introduced BiFPN as a weighted bidirectional feature-fusion strategy and demonstrated its effectiveness as a compact default for multi-scale detectors under tight compute budgets [17]. EfficientDet’s BiFPN, in particular, established weighted bidirectional fusion as a strong default for compact multi-scale detectors.

### 2.4. Small-Defect Detection, Attention, and Loss Shaping

Small defects demand extra care. Recent online weld defect detectors explicitly report pronounced gains on small-scale porosity-type defects by introducing attention-enhanced heads and shape-aware IoU losses to strengthen localization and convergence [9,10]. Scale-aware attention and dynamic cross-scale feature interaction are common remedies when shallow-layer details get drowned out by high-level semantics. Attention modules such as CBAM and dual-attention designs help emphasize discriminative cues and suppress background-dominant responses. Representative attention modules include CBAM and dual-attention designs that jointly model channel/spatial dependencies to emphasize discriminative cues and suppress background-dominant activations [18,19]. On the training side, dense detection benefits from losses designed for imbalance and localization quality. Focal loss, generalized focal loss, and shape-aware IoU losses have all been used to tighten box regression and stabilize training. Focal loss and generalized focal loss are widely adopted to mitigate class imbalance and incorporate localization quality into training, improving stability for rare defect categories [20,21]. Meanwhile, shape-aware IoU metrics provide more informative regression signals for irregular or elongated targets and have shown benefits in dense small-target settings as well [22].

Where this work sits:

This paper focuses on RGB visual inspection of galvanized-sheet MIG seams under reflective and texture-rich conditions and explicitly frames the task as five-class detection with an ROI around the seam. We refine YOLOv10n with small-defect-oriented feature enhancement, BiFPN fusion, and prediction-stage refinement, aiming for a model that survives contact with shop-floor constraints rather than only scoring well in a sandbox. Accordingly, our design choices follow the recent trend of lightweight, attention-augmented, multi-scale detectors for weld inspection under complex textures and reflective conditions, while explicitly targeting edge-ready deployment constraints [8,9,10,17].

## 3. Method

**Figure 1:**
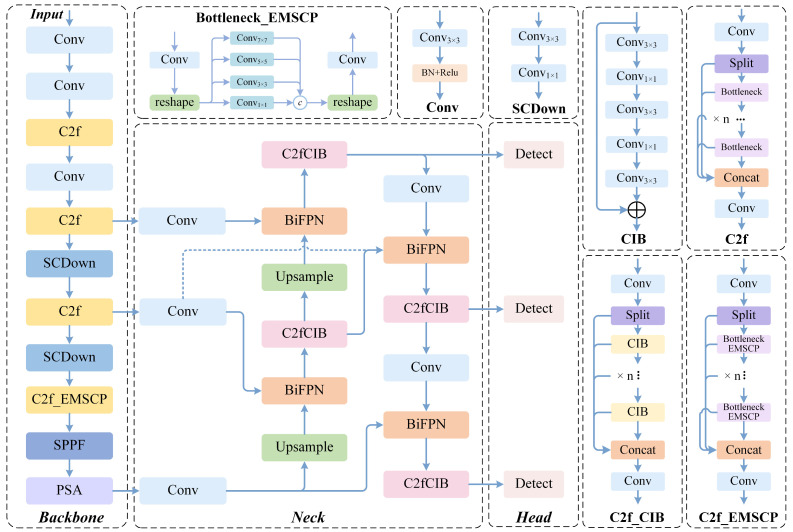
YOLOv10n-MIG Training and Detection Workflow.

YOLO-MIG follows a standard one-stage detection pipeline, in which the backbone extracts features, the neck performs multi-scale feature fusion, and the head predicts categories and bounding boxes (Figure 1). Recent weld-inspection studies have shown that YOLO-style detectors provide a practical accuracy–latency trade-off for online defect localization, and many lightweight variants improve robustness through targeted feature enhancement and refinement modules [23,24,25]. Similar real-time constraints also arise in in situ monitoring of arc- or energy-deposition manufacturing, where CNN- and YOLO-based models are used to detect or segment melt-pool states at production speed, further motivating compact designs deployable near the process [26,27]. In this work, the original YOLOv10n training and inference framework is retained, while the components most sensitive to weld-scene interference are refined, particularly those related to weak small-defect representation and background-induced false activations. A persistent challenge in weld vision is the specular nature of molten metal together with strong arc-light interference, both of which can distort appearance cues and make stable feature extraction and decision formation difficult in practice [28,29,30,31].

### 3.1. C2f-EMSCP Backbone Network

**Figure 2:**
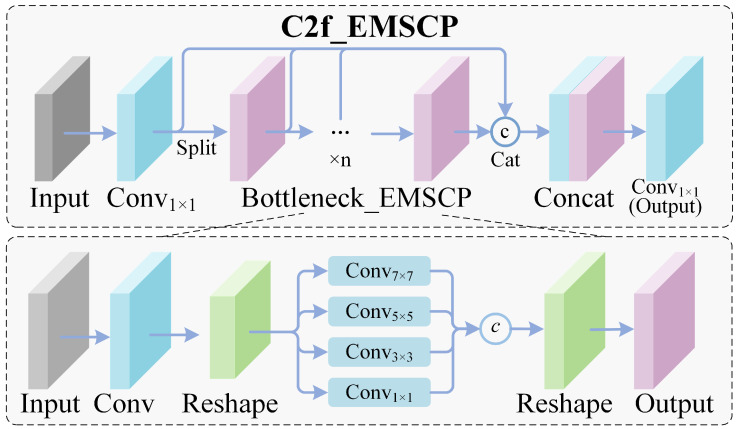
Structure of the proposed C2f-EMSCP block and its integration into the YOLO-MIG backbone.

Although C2f modules (Figure 2) are computationally efficient, weld defects often provide only weak and limited texture information. Under reflective lighting conditions, subtle defect cues may be lost during downsampling. This is consistent with weld-pool vision and penetration-related studies, where specular reflections and arc radiation make low-contrast structures hard to capture reliably from raw imagery [28,29,30,31]. We replace the final C2f stage with C2f-EMSCP, which is designed to strengthen fine-grained defect cues through multi-scale enhancement and channel reweighting without substantially increasing the parameter count. Related lightweight weld defect detectors often introduce dedicated multi-scale reinforcement blocks to enrich defect cues while keeping computational cost low, which supports our choice of a defect-oriented enhancement design in the backbone [23,24]. Our multi-scale enhancement can be written as a lightweight multi-branch transformation that expands the receptive field without a heavy parameter increase. Given an input feature map FFF, we first compute parallel-scale responses and concatenate them:

(1) $$F_{\mathrm{ms}}=\mathrm{Concat}(\phi_{1\times 1}(F),\;\phi_{3\times 3}(F),\;\phi_{5\times 5}\left(F)\right)$$

The concatenated features are then compressed and combined with a residual shortcut to preserve low-level detail:

(2) $$F_{\mathrm{enh}}=\phi_{1\times 1}(F_{\mathrm{ms}})+F$$

This design is intentionally lightweight: it enhances subtle defect patterns while keeping the module compact enough for edge deployment. Scale-aware modeling and attention have been repeatedly shown to help in small-defect settings, so the design choice is not exotic—it is pragmatic. In particular, attention-augmented YOLO variants for weld imagery report improved small-target sensitivity and reduced misjudgment by emphasizing informative channels/regions under complex backgrounds [24,25].

At the tail of the backbone, we append a PSA-style attention block to improve global context modeling. Such context-aware attention is particularly helpful when seam edges, spatters, and highlights resemble defect patterns, since robust seam perception under strong arc radiation has been shown to benefit from stronger global/context cues rather than purely local responses [32]. We express this global-context step with a compact self-attention formulation. Specifically, we project the feature map to query, key, and value tensors and compute attention weights via scaled dot-product:

(3) $$s\;=\;\sigma (W_{2}\;\delta (W_{1}\;\mathrm{GAP}(F_{\mathrm{enh}}))),\;F^{\prime }\;=\;s⊙F_{\mathrm{enh}}$$

In practice, this mechanism helps the network distinguish weak defect patterns from seam highlights and other background interference. Attention-based mechanisms are widely used for this type of discrimination under cluttered visual conditions.

### 3.2. BiFPN Neck Network

**Figure 3:**
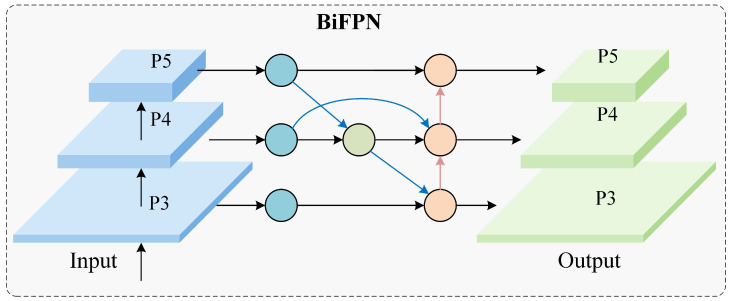
BiFPN neck used in YOLO-MIG for bidirectional multi-scale feature fusion.

Feature fusion is a stage at which small-defect information is often lost. A one-way top-down pyramid may dilute shallow-layer details that are essential for small-target detection. We use BiFPN (Figure 3), which fuses features bidirectionally and learns fusion weights during training. In BiFPN, each fusion node aggregates multi-scale inputs using learnable non-negative weights, so that the network can adaptively emphasize the most informative resolution under reflective and texture-rich conditions. Concretely, given a set of input feature $\{F_{i}{\}}_{i=1}^{N}$, the fused representation is computed:

(4) $$\hat{F}=\frac{\sum_{i=1}^{N}w_{i}F_{i}}{\sum_{i=1}^{N}w_{i}+\varepsilon },\;w_{i}=\mathrm{ReLU}(\alpha_{i})$$

where $w_{i}$ are trainable weights constrained to be non-negative (implemented with a ReLU), and ε prevents numerical instability. This weighting mechanism helps preserve weak small-defect cues that might otherwise be diluted by uniform feature aggregation. BiFPN was originally introduced as a weighted bidirectional pyramid for efficient multi-scale fusion, and subsequent works have adopted or modified BiFPN-style designs to strengthen small-object detection under resource constraints across multiple domains [33,34,35]. This design enables the model to preserve local detail while still benefiting from higher-level semantic information.

### 3.3. C2fCIB Head Network


(5) $$G=\sigma (\phi_{3\times 3}(F_{h})),\;F_{clean}=G⊙F_{h}$$

(6) $$\hat{y},\hat{b}=\mathrm{Head}(F_{\mathrm{clean}})$$

**Figure 4:**
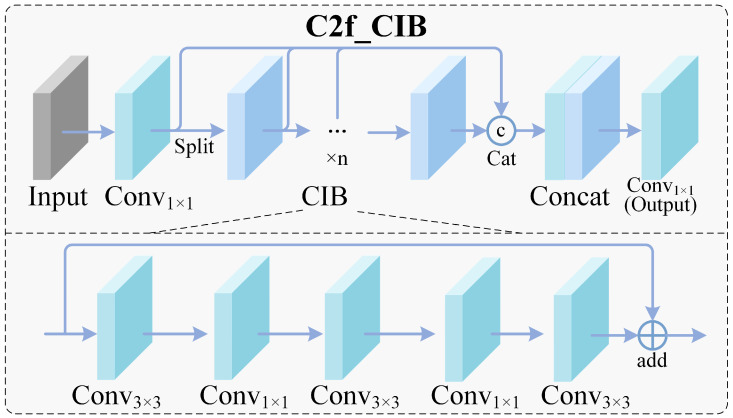
C2fCIB module and the modified YOLO-MIG detection head.

In weld-seam imagery, false positives frequently occur around seam boundaries, texture transitions, and illumination artifacts. In weld defect detection, explicitly suppressing interference information (e.g., seam-structure distractions and background noise) has been shown to significantly reduce both missed detections and false alarms in practical imaging conditions [23,24,25]. We introduce C2fCIB modules (Figure 4) into the detection head to refine prediction features and suppress these background-driven activations. Accordingly, we treat head refinement as a lightweight post-fusion filtering step that complements backbone enhancement and BiFPN fusion, improving prediction stability without introducing heavy latency [17,24,25]. We implement this refinement as a lightweight gating operation that learns to suppress response patterns correlated with seam boundaries and illumination artifacts. Given head features $F_{h}$, a spatially aware gate G is predicted and applied as:

(7) $$G=\sigma (\phi_{3\times 3}(F_{h})),\;F_{\mathrm{clean}}=G⊙F_{h}$$

The cleaned features $F_{\mathrm{clean}}$ are then used for final classification and box regression. This simple mechanism can effectively reduce edge-following false positives without introducing noticeable latency. Functionally, it serves as a lightweight prediction-refinement stage that improves reliability with limited computational overhead, which is important for stable deployment in practical industrial settings.

## 4. Experiment

### 4.1. Dataset Construction

We collected a galvanized-sheet MIG weld dataset in a real welding workshop using an industrial camera (Daheng Imaging, Beijing, China). In real workshops, weld-surface imaging is frequently affected by specular highlights, non-uniform illumination, and reflection-induced pseudo-defects, which can substantially reduce the stability of vision-based inspection if not explicitly considered in data collection and model design [1,5,6,7,8,15,16,36,37,38].

**Figure 5:**
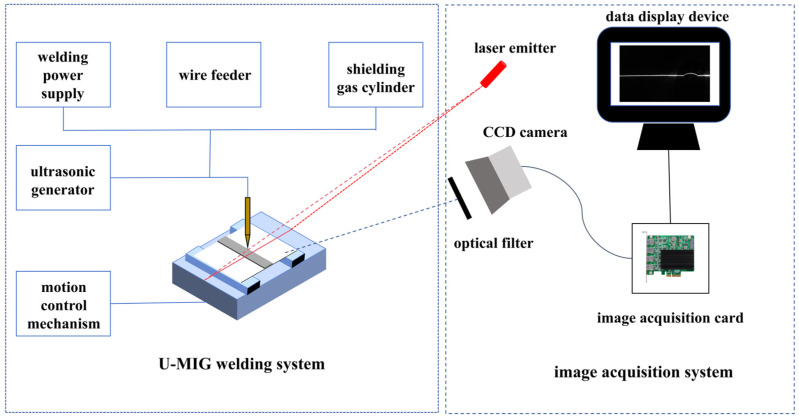
Weld seam image acquisition system.

We collected a galvanized-sheet MIG weld dataset in a real welding workshop using an industrial CCD camera equipped with a suitable imaging lens. The weld seam image acquisition system used in this study is shown in Figure 5.

Defects occur in and near the seam, so we focus detection on a weld-seam ROI. Using a seam-centered ROI is consistent with practical vision inspection pipelines, where restricting the search region helps mitigate background clutter and illumination artifacts while improving defect separability [32,37]. This mirrors the way real inspection systems operate: they constrain the search space rather than asking the model to fight the entire frame.

**Figure 6:**
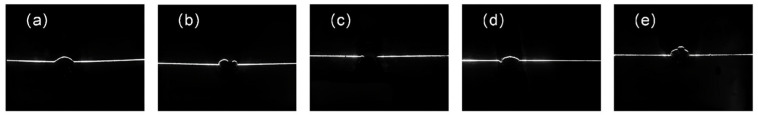
(**a**–**e**) Structured-light images of weld-bead surface quality: (**a**) good, (**b**) blowhole, (**c**) burn-through, (**d**) undercut, (**e**) embossment.

To accurately and effectively acquire informative features of weld defects, this study employed a line-structured-light-assisted imaging approach. Based on the principle of optical triangulation, a line laser was projected onto the weld surface, and the reflected light stripe was captured by an imaging device. This method enables a more precise representation of surface-defect characteristics. By collecting and analyzing line-structured-light images of different weld types, a dedicated database for welding-defect detection was established. Five common surface-quality categories for galvanized steel sheets were defined, including good, blowhole, burn-through, undercut, and embossment, with the corresponding line-structured-light images shown in Figure 6.

**Figure 7:**
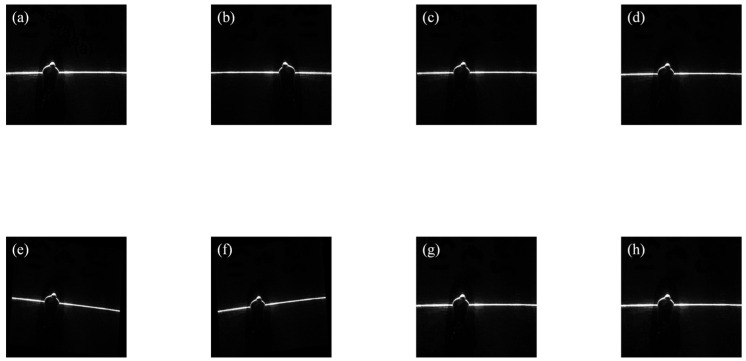
Data augmentation methods: (**a**) Original image, (**b**) Horizontal flip, (**c**) Noise addition, (**d**) Contrast enhancement, (**e**) 5° clockwise rotation, (**f**) 5° counterclockwise rotation, (**g**) 5 mm upward shift, (**h**) 5 mm downward shift.

The original dataset consisted of 326 workshop-acquired images collected at the workpiece level. To improve training diversity, only the training subset was augmented, whereas the validation and test subsets consisted exclusively of original, non-augmented images. Although augmentation increases sample diversity and helps reduce overfitting, it cannot fully replace the natural variability of independent real industrial samples. In deep learning, data augmentation involves modifying the input data through simple operations such as translation, scaling, and contrast enhancement, which generate new training samples. These operations do not alter the image class but increase the number of training samples, enhancing the model’s generalization capability and performance. Additionally, they help reduce over-reliance on specific features and prevent overfitting. The collected images underwent data augmentation, including rotation, translation, noise addition, and contrast enhancement. The specific augmentation methods and their corresponding result images are shown in Figure 6. This process expanded the original dataset to 2608 images, with 554 representing good weld seams, 546 showing burn-through defects, 488 displaying undercut defects, 542 depicting blowhole defects, and 478 illustrating embossment defects (Table 1). The original images were first divided at the workpiece level into training, validation, and test subsets before any data augmentation was applied. Only the training subset was augmented (Figure 7), whereas the validation and test subsets consisted exclusively of original, non-augmented images. Therefore, augmented samples derived from a given original image were never allowed to appear across different subsets, which avoids data leakage between training and evaluation. To reduce overly optimistic evaluation caused by highly correlated seam segments captured from the same workpiece, we split the dataset at the workpiece level rather than at the image level [1,11].

The final evaluation was performed only on the original validation and test images.

We augment only the training set using mosaic-style composition and standard geometric/photometric perturbations to improve generalization. Training-set augmentation is adopted to improve generalization to small and low-contrast defects under varying textures and lighting, which is also a common practice in recent YOLO-based weld defect detection studies [9,10,11,23,24,25].

### 4.2. Experimental Environment and Parameter Settings

We run experiments on an Intel Core i7-1250P CPU with an NVIDIA GeForce MX550 GPU (4 GB VRAM) and 16 GB RAM (Table 2). Such a modest GPU budget is representative of edge or near-line deployment constraints reported in recent weld-inspection surveys and online monitoring systems, where lightweight models are preferred over data-center-scale backbones [37,38]. We use Windows 11, Python 3.9, PyTorch 2.6, and CUDA 12.5. We resize images to 640 × 640 and train with SGD and cosine annealing for up to 200 epochs (Table 3), enabling early stopping (patience = 50). Batch size is 1 due to VRAM limits; such a constrained GPU setting is consistent with recent studies that evaluate YOLO-based defect detectors on embedded or resource-limited NVIDIA devices, where deployment optimizations such as TensorRT export and reduced-precision inference (e.g., FP16/INT8) are commonly adopted to balance speed and accuracy [39]. In line with these edge-deployment practices, we report FP16 weight size alongside parameter count and GFLOPs to better reflect real-world deployment costs [40,41]. To keep the comparison fair, we apply the same training protocol to all models. Such resource-constrained settings reflect realistic deployment conditions, where lightweight CNN/YOLO models are preferred for online welding monitoring and inspection close to the production line [1,2,23,24,26,27]. The model is trained with a standard detection objective that combines localization, classification, and distribution-based box refinement:

(8) $$L=\lambda_{\mathrm{box}}L_{\mathrm{iou}}+\lambda_{\mathrm{cls}}L_{\mathrm{cls}}+\lambda_{\mathrm{dfl}}L_{\mathrm{dfl}}$$

where $L_{\mathrm{iou}}$ measures bounding-box overlap, $\lambda_{\mathrm{cls}}$ is the classification loss, and $\lambda_{\mathrm{dfl}}$ denotes distribution focal loss for more stable regression on small targets.

### 4.3. Evaluation Metrics

We report mAP@0.5 and mAP@0.5:0.95, plus precision and recall. To make the detector’s behavior interpretable at the shop-floor level, we report precision and recall, defined by the ratio of true positives to predicted positives and to ground-truth positives, respectively:

(9) $$\mathrm{Precision}=\frac{\mathrm{TP}}{\mathrm{TP}+\mathrm{FP}}$$

(10) $$\mathrm{Recall}=\frac{\mathrm{TP}}{\mathrm{TP}+\mathrm{FN}}$$

These two measures are particularly helpful when false alarms cluster around seam edges or highlights, while missed detections tend to occur on tiny, low-contrast defects. Following standard detection practice, we summarize performance over the full precision–recall curve using average precision (AP) for each class and mean AP (mAP) across all classes:

(11) $${\mathrm{AP}}_{c}=\int_{0}^{1}P_{c}(r)\;\mathrm{dr}$$

(12) $$\mathrm{mAP}=\frac{1}{C}{\sum_{c=5}^{C}\mathrm{AP}}_{c}$$

In our setting, the class set includes four defect categories and a “good” operational label, so we additionally inspect per-class AP to avoid over-reading a single aggregate number. This practice is also aligned with recent real-time defect detection work targeting edge deployment, where class imbalance and performance–efficiency trade-offs must be analyzed beyond a single aggregated metric [40,41]. Because industrial weld datasets are often imbalanced (normal/good samples dominate while defect samples are relatively scarce), we report per-class AP, a normalized confusion matrix, and typical failure patterns in addition to overall mAP to avoid misleading conclusions [1,11]. This is a common issue in industrial inspection, where “normal” data often overwhelm rare defects.

### 4.4. Ablation Experiment

We conduct ablation experiments by enabling one module at a time and then combining all modules into the final model (Table 4). Ablation experiments are commonly used in improved YOLO-based weld defect detectors to quantify the individual contributions of feature-fusion and refinement strategies under a unified training protocol [9,10,23,24,25]. Training curves of precision/recall/mAP and loss terms are shown in Figure 5 and Figure 6, respectively.

### 4.5. Comparison with Detection Baselines

**Figure 8:**
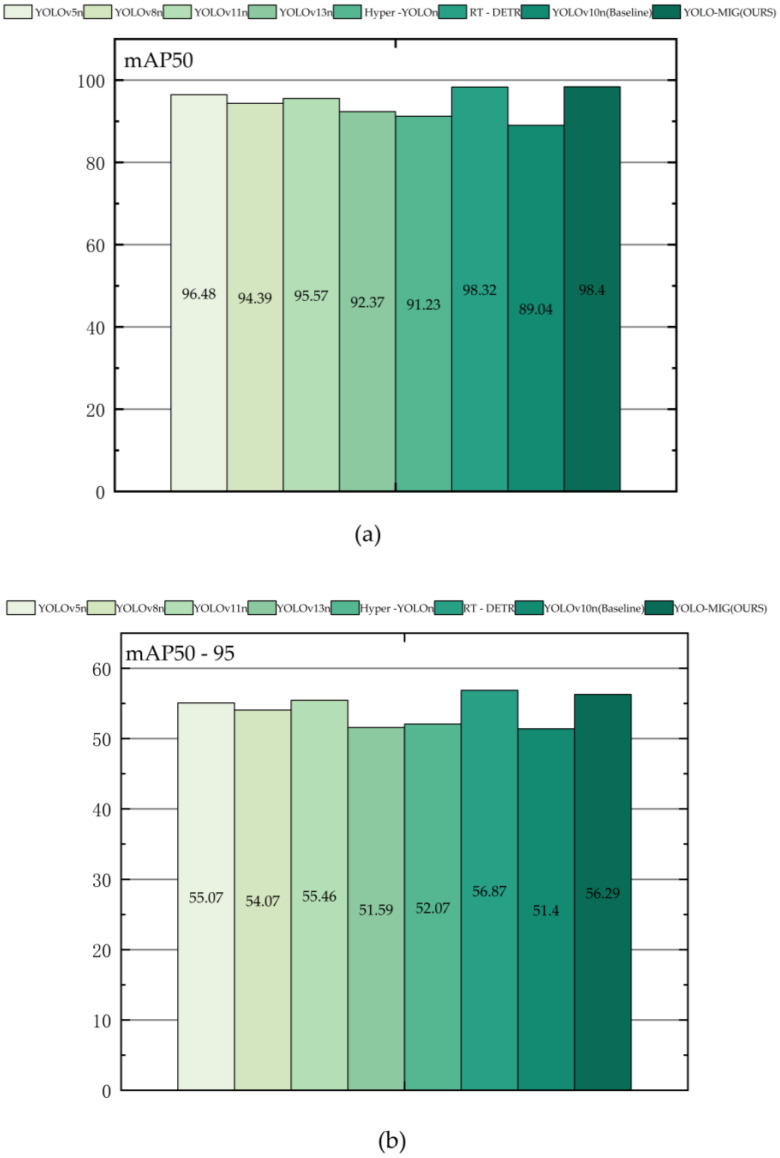

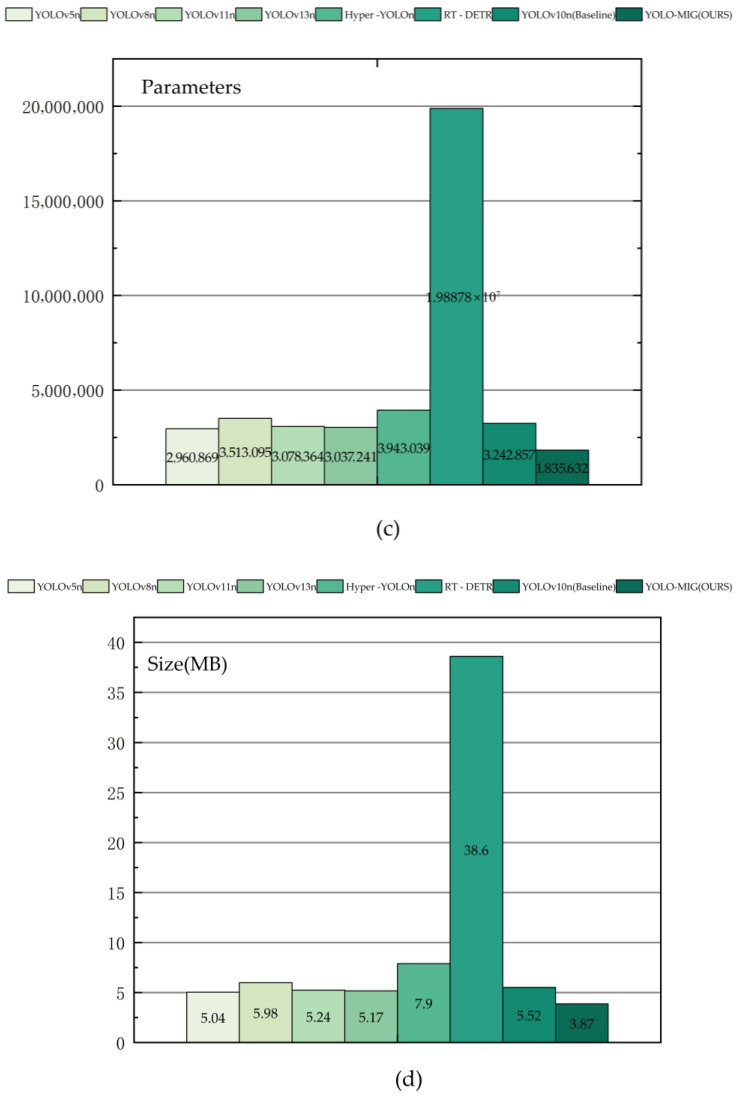
(**a**–**d**) Ablation study of YOLOv10-based defect detection model for galvanized sheet MIG welding.

To assess YOLO-MIG under a controlled protocol, we keep the comparison consistent: the same data split, the same input resolution (640 × 640), and the same evaluation code. Following common practice in weld defect detection research, all compared models are trained and evaluated under the same split, input resolution, and evaluation pipeline to ensure a fair comparison of accuracy–efficiency trade-offs [1,9,23,24]. What changes is the network design. We compare the YOLOv10n baseline with three single-module variants, full model, five YOLO and ablation variants and RT-DETR. Table 5 reports quantitative results, and Figure 8 shows representative detections on real workshop images.

The headline result is clear. YOLO-MIG reaches 98.4% mAP@0.5 and 56.29% mAP@0.5:0.95 on the test set, while staying compact (1.83 M parameters and 3.87 MB FP16 weights). These numbers matter in our setting because the model is intended to run close to the production line, where compute budgets are rarely generous. With an NVIDIA MX550, YOLO-MIG maintains stable real-time inference, indicating that the accuracy gain does not come from an impractically heavy network.

The ablations also help isolate where the improvements originate. The C2f-EMSCP backbone improves sensitivity to subtle cues that are easily drowned out by reflective backgrounds, which is especially relevant for small blowholes. The BiFPN neck improves cross-scale feature reuse, which helps retain small-target information that can be diluted in one-way top-down fusion. The C2fCIB head plays a practical “cleanup” role by reducing spurious activations around seam edges and texture transitions, improving prediction-stage discrimination without a large latency penalty. False alarms around seam edges and highlight regions have been widely observed in reflective metal inspection, motivating either highlight-aware preprocessing or prediction-stage feature refinement to suppress reflection-induced activations [36,38]. When these components are integrated their effects reinforce one another, and the full model provides the strongest balance between accuracy and efficiency.

**Figure 9:**
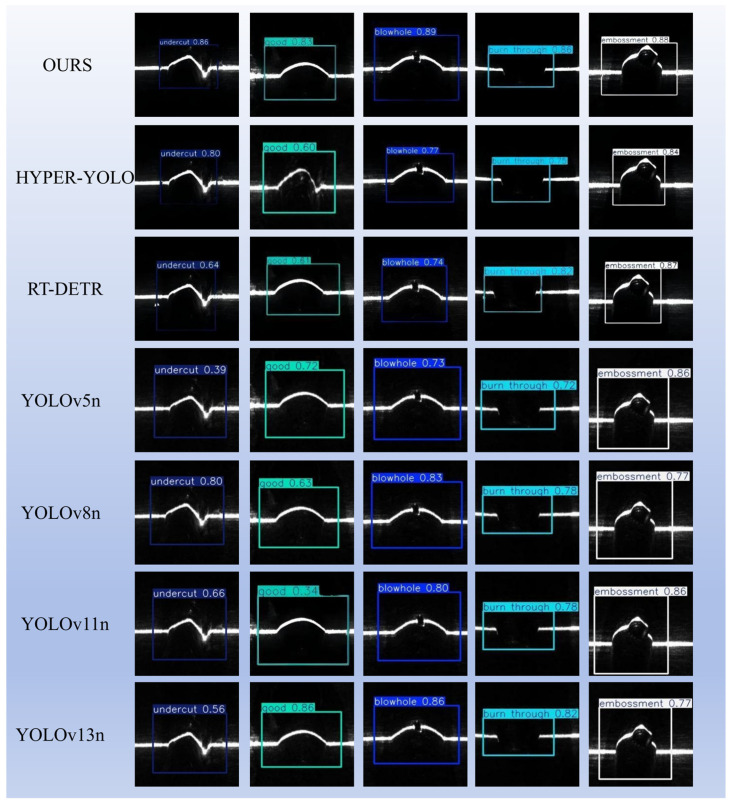
Visualization of industrial surface defect detection results (based on NVIDIA MX550).

Table 5 also lists several common classification backbones (Hyper-YOLOn [39], and RT-DETR). Considering that recent welding defect studies have also adopted classification- or transfer-learning-based frameworks, we additionally included a ResNet-based comparative experiment to broaden the methodological comparison [42]. They are reported as model-scale references (parameters and FP16 weight size), not as detection baselines. Moreover, results from different inspection modalities (e.g., X-ray radiographs versus RGB seam images) or different task formulations (classification backbones versus detection heads) are not directly comparable in mAP unless embedded into the same detection framework and evaluated under identical protocols [1,4,23,25]. Therefore, the quantitative detection comparison in this study relies on YOLOv10n and the ablation variants as the primary baselines. The visualization results of industrial surface defect detection are presented in Figure 9.

**Figure 10:**
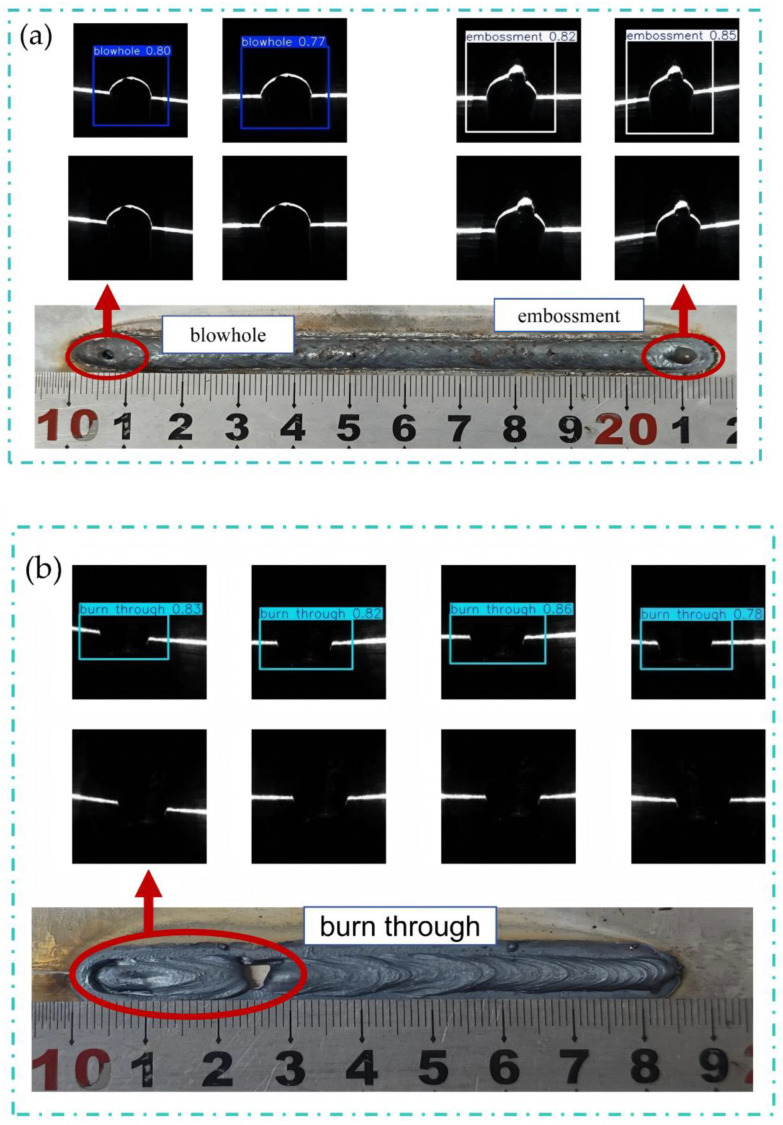

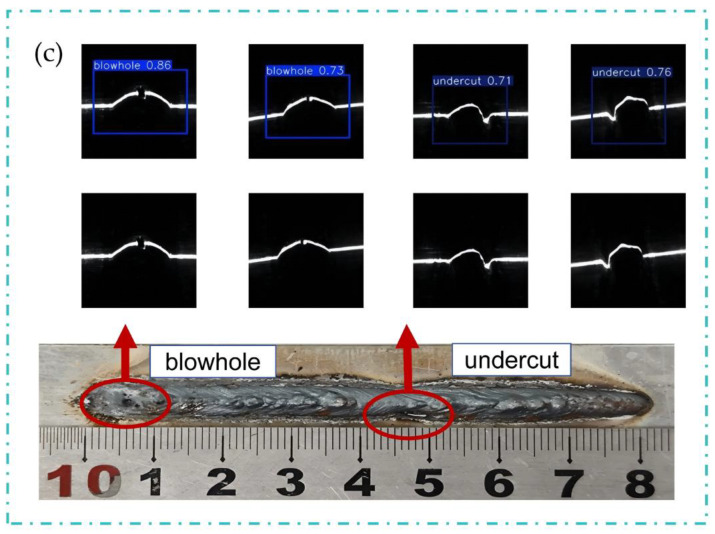
(**a**–**c**) Representative examples of actual detection results.

Based on the improved YOLOv10 model, defect detection was performed on the test set, and representative detection results are shown in Figure 10a–c.

### 4.6. Confusion-Matrix

**Figure 11:**
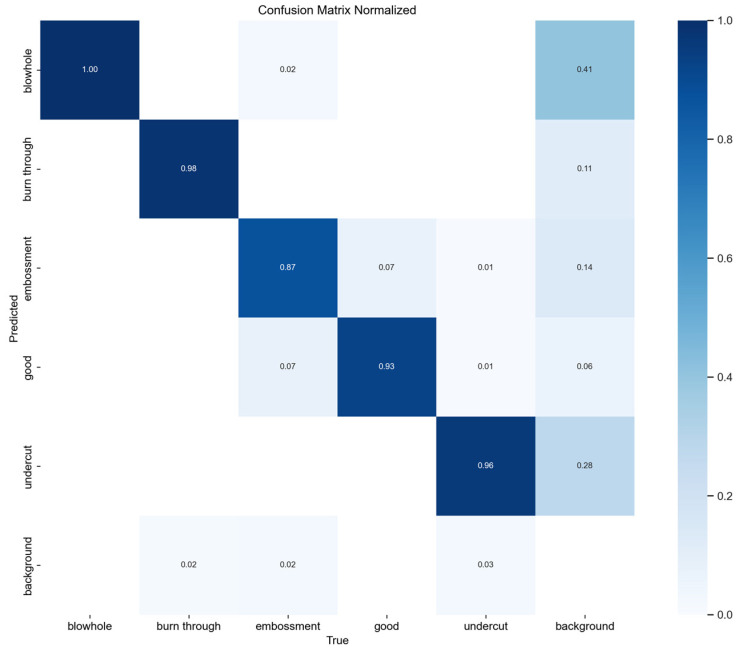
Normalized confusion matrix of YOLO-MIG on the original test set.

Figure 11 presents the normalized confusion matrix of YOLO-MIG on the original test set. Although the model achieves strong overall performance, several typical error patterns remain. Missed detections mainly occur for very small blowholes and shallow undercuts with weak local contrast, especially when defect boundaries are partially masked by reflected structured-light stripes or seam texture. False positives are more likely near seam edges, highlight regions, and texture transitions, where background patterns may resemble defect cues. In addition, some borderline ‘good’ samples may be confused with weak embossment- or undercut-like structures. These results indicate that the remaining errors are primarily associated with small target size, low contrast, and reflection-induced interference.

## 5. Conclusions

YOLO-MIG targets a very practical question: can we detect weld defects on a galvanized-sheet MIG line with a model that fits edge hardware and still behaves sensibly under messy lighting and texture? We frame the task as five-class detection—burn-through, undercut, blowhole, embossment, and good—and refine YOLOv10n where it tends to struggle on weld seams. We strengthen weak defect representation in the backbone, improve cross-scale alignment with BiFPN, and refine the head to reduce background-driven false alarms. These design choices follow the broader trend of improving small-defect sensitivity and suppressing background interference in real-world weld inspection by combining multi-scale fusion and attention-style feature filtering [1,9,10,23,24,25].

On a workshop-collected dataset of 2608 labeled images, the model achieves strong mAP while remaining compact enough for FP16 edge deployment. In practice, deploying the detector close to the line enables timely feedback and reduces the cost and latency of streaming high-resolution imagery to centralized servers [1,2,26,27]. For a shop-floor system, that balance is the difference between a promising prototype and something you can actually run next to the line.

The present study still has several limitations that are important for practical industrial deployment. Although the proposed model performs well on the current galvanized-sheet MIG dataset, the limited number of original images (326) remains an important constraint on robustness and generalization. Data augmentation improves training diversity, but it cannot fully substitute for independent real-world samples collected under broader production conditions. Different welding processes may lead to variations in weld morphology, defect appearance, and background texture, while different materials may exhibit distinct surface reflectance characteristics. In addition, changing illumination conditions and reflection patterns in real workshop environments may further affect detection robustness. Sensor variability, process-parameter fluctuations, and environmental complexity may also introduce domain shift between training and deployment scenarios. Therefore, further validation under more diverse production environments is still necessary.

Therefore, a broader cross-domain benchmark and a more detailed failure analysis—such as which defect categories are more likely to be missed, which background cues tend to cause false positives, and how the “good” category behaves in borderline cases—would further strengthen the reproducibility and practical relevance of the conclusions. Promising future directions include cross-line and cross-condition validation, data rebalancing or synthesis for rare defects, and adaptation strategies for improving robustness from controlled experimental settings to real shop-floor environments [1,11]. As emphasized in recent surveys, robustness under distribution shifts remains one of the main barriers from laboratory studies to industrial deployment, and we do not claim that the present work has fully resolved this issue.

## Figures and Tables

**Table 1:** Dataset split before augmentation and final training-set size after augmentation.

Porosity Size	Training Set	Validation Set	Test Set	Total
Burn-through	478	45	23	546
Undercut	427	41	20	488
Blowhole	474	45	23	542
Embossment	418	40	20	478
Good	485	46	23	554
Total	2282	217	109	2608

**Table 2:** Hardware and software environment.

Component	Specification
CPU	Intel Core i7-1250P
GPU	NVIDIA GeForce MX550
RAM	16 G
VRAM	4 G
Operating System	Windows 11
CUDA version	12.5
Programming Language	Python 3.9
Framework	Pytorch 2.6
Development Environment	Pycharm 2022.1.4

**Table 3:** Training hyperparameters.

Hyperparameters	Values
Image size	640 × 640
Batch-size	1
Optimizer	SGD
Patience	50
Epochs	200

**Table 4:** Ablation configuration. (In the following table, √ indicates that the module is applied, while × indicates that the module is not applied).

Model	C2f-EMSCP	BiFPN	C2fCIB
YOLOv10n (Baseline)	×	×	×
YOLOv10n-C2f-EMSCP	√	×	×
YOLOv10n-BiFPN	×	√	×
YOLOv10n-C2f-CIB	×	×	√
YOLO-MIG	√	√	√

**Table 5:** Performance comparison of YOLO-MIG with YOLO and ablation variants on the test set. (All models in Table 5 were trained and evaluated on our dataset using the same data split, input resolution, training epochs, and hyperparameter settings for a fair comparison).

Model	mAP50 (%)	mAP50-95 (%)	Parameters	Size (MB)	GFLOPs
YOLOv5n	96.48	55.07	2,960,869	5.04	7.7
YOLOv8n	94.39	54.07	3,513,095	5.98	8.7
YOLOv11n	95.57	55.46	3,078,364	5.24	6.5
YOLOv13n	92.37	51.59	3,037,241	5.17	6.4
Hyper-YOLOn	91.23	52.07	3,943,039	7.9	10.8
RT-DETR	98.32	56.87	19,887,780	38.6	57.2
YOLOv10n (Baseline)	89.04	51.40	3,242,857	5.52	9.2
YOLOv10n-C2f-EMSCP	96.4	55.73	2,943,286	4.57	7.81
YOLOv10n-BiFPN	91.37	53.59	3,221,757	5.24	6.44
YOLOv10n-C2f-CIB	89.54	54.29	1,876,423	4.14	7.44
YOLO-MIG (OURS)	98.4	56.29	1,835,632	3.87	7.37
Res_WS_S4	94.14	55.93	11,127,000	21.48	28.45

## Data Availability

The data presented in this study are available on request from the corresponding author. The data are not publicly available due to privacy.
